# Cross-Cultural Examination of Links between Parent–Adolescent Communication and Adolescent Psychological Problems in 12 Cultural Groups

**DOI:** 10.1007/s10964-020-01212-2

**Published:** 2020-03-12

**Authors:** Sabina Kapetanovic, W. Andrew Rothenberg, Jennifer E. Lansford, Marc H. Bornstein, Lei Chang, Kirby Deater-Deckard, Laura Di Giunta, Kenneth A. Dodge, Sevtap Gurdal, Patrick S. Malone, Paul Oburu, Concetta Pastorelli, Ann T. Skinner, Emma Sorbring, Laurence Steinberg, Sombat Tapanya, Liliana Maria Uribe Tirado, Saengduean Yotanyamaneewong, Liane Peña Alampay, Suha M. Al-Hassan, Dario Bacchini

**Affiliations:** 1 University West, Trollhättan, Sweden; 2 Duke University, Durham, NC USA; 3 University of Miami Miller School of Medicine, Mailman Center for Child Development, Miami, FL USA; 4 Institute for Fiscal Studies, London, UK; 5 Eunice Kennedy Shriver National Institute of Child Health and Human Development, Bethesda, MD USA; 6 University of Macau, Macau, China; 7 University of Massachusetts, Amherst, MA USA; 8 Università di Roma “La Sapienza”, Rome, Italy; 9 Maseno University, Maseno, Kenya; 10 Temple University, Philadelphia, PA USA; 11 Chiang Mai University, Chiang Mai, Thailand; 12 Universidad San Buenaventura, Medellín, Colombia; 13 Ateneo de Manila University, Quezon City, Philippines; 14 Hashemite University, Zarqa, Jordan; 15 Emirates College for Advanced Education, Abu Dhabi, UAE; 16 University of Naples “Federico II”, Naples, Italy

**Keywords:** Parent-adolescent communication, Adolescent secrecy, Psychological problems, Universal parenting

## Abstract

Internalizing and externalizing problems increase during adolescence. However, these problems may be mitigated by adequate parenting, including effective parent–adolescent communication. The ways in which parent-driven (i.e., parent behavior control and solicitation) and adolescent-driven (i.e., disclosure and secrecy) communication efforts are linked to adolescent psychological problems universally and cross-culturally is a question that needs more empirical investigation. The current study used a sample of 1087 adolescents (*M* = 13.19 years, *SD* = 0.90, 50% girls) from 12 cultural groups in nine countries including China, Colombia, Italy, Jordan, Kenya, Philippines, Sweden, Thailand, and the United States to test the cultural moderation of links between parent solicitation, parent behavior control, adolescent disclosure, and adolescent secrecy with adolescent internalizing and externalizing problems. The results indicate that adolescent-driven communication, and secrecy in particular, is intertwined with adolescents’ externalizing problems across all cultures, and intertwined with internalizing problems in specific cultural contexts. Moreover, parent-driven communication efforts were predicted by adolescent disclosure in all cultures. Overall, the findings suggest that adolescent-driven communication efforts, and adolescent secrecy in particular, are important predictors of adolescent psychological problems as well as facilitators of parent–adolescent communication.

## Introduction

Early adolescence is a period of elevated risk for the development of psychological problems. Internalizing problems (such as anxiety and depression symptoms) as well as externalizing problems (such as conduct problems, aggression, and hyperactivity) are the most common child psychological health problems and often onset in early adolescence (Merikangas et al. 2010). Although generally early adolescent boys exhibit more externalizing problems, whereas early adolescent girls exhibit more internalizing problems, girls tend to show greater increases in overall psychological problems during early adolescence (Reitz et al. 2005). However, experiencing these problems in early adolescence may have life-long consequences for adolescents’ future regardless of an adolescent’s gender (Reinke et al. 2012). Numerous studies have examined how parents might prevent these problems from arising. Research suggests that parent-driven communication efforts are protective of adolescent functioning (Racz and McMahon 2011). Specifically, when parents solicit information from their adolescents about where adolescents are and what they are doing (i.e., parent solicitation) and set rules and limits for appropriate adolescent behavior (parent behavioral control), adolescents tend to show more positive developmental outcomes (Fletcher et al. 2004). Although this work has greatly enhanced understanding of the protective effects of parent communication on adolescent mental health, several gaps remain in existing literature. One gap is that previous literature often assumes a unidirectional link from parent-driven communication efforts to adolescent functioning (Dishion and McMahon 1998) while the possible effects from adolescent communication efforts and behavior to parenting are not considered. A second gap is that, although some studies acknowledge the adolescent as an actor in parent–adolescent communication through adolescents’ own disclosure of information (Stattin and Kerr 2000), most studies fail to examine the varying effects of different types of adolescent-driven communication strategies. A third gap is that the examination of cultural differences in parent-initiated and adolescent-driven communication strategies is scarce. This study examines the reciprocal associations between parent-driven (parent solicitation and behavior control) and adolescent-driven (adolescent disclosure and secrecy) communication efforts and adolescent psychological problems (internalizing and externalizing). Moreover, the study uses a cross-cultural sample of adolescents from 12 different cultural contexts to investigate how potential links between parent–adolescent communication and adolescent psychological problems are moderated by culture.

### Parenting Adolescents

Parents shape their adolescents’ development through their own parenting practices (Darling and Steinberg 1993; Soenens et al. 2019). Such practices include parent-driven communication strategies such as parent solicitation and behavioral control (Dishion and McMahon 1998). Parents can obtain information about adolescents’ everyday lives by initiating conversation with their adolescent, by asking questions to adolescents themselves, or by talking to their adolescents’ friends and parents of their friends (parent solicitation; Bornstein et al. 2013). Parents also communicate rules of behavior and control adolescents’ freedom to come and go as they please (parent behavior control; Stattin and Kerr 2000). Ideally, asking adolescents for information or communicating rules of behavior should help parents stay involved in their adolescents’ lives and help parents protect their adolescents from harm. However, traditional gender roles are often reflected in the interaction between parents and their adolescents, with parents communicating different behavioral expectations for girls and boys (Leaper and Farkas 2015). Indeed, early adolescent girls report higher levels of parent behavior control and solicitation than their male counterparts (Kapetanovic et al. 2017). In addition, studies suggest that parents with higher socioeconomic status, often measured by parent educational level, generally are more supportive and have more knowledge of their adolescents’ activities than parents with low socioeconomic status (Morawska et al. 2009). Despite these relative differences in parenting practices with regards to child gender and family socioeconomic status, meta-analyses indicate that parent-driven communication efforts are related to lower levels of internalizing (Pinquart 2017b) and externalizing problems (Pinquart 2017a) regardless of adolescent gender or parent education (Akcinar and Baydar 2014). Importantly, however, some studies have suggested that parent solicitation may be related to poorer adolescent developmental outcomes, including internalizing (Hessel et al. 2017) and externalizing problems (Garthe et al. 2015), because such parenting practices relate to perceived privacy invasions in adolescents (Hawk et al. 2008) and feelings of being overly controlled by parents (Kapetanovic et al. 2017), particularly in boys (Hawk et al. 2008). Therefore, parental active efforts of communication could be protective against the development of adolescent psychological problems, but only if adolescents do not perceive them as intrusive.

### Adolescents’ Communication Strategies and Information Management

Another way for parents to obtain knowledge of their adolescents’ everyday lives is through adolescents’ own use of communication strategies and consequent management of information. Two decades ago, Stattin and Kerr (2000) (see also Kerr and Stattin 2000) called for a reconceptualization of parental communication and monitoring efforts, bringing attention to the agency of the child in the parent–adolescent relationship. They suggested that parents mainly obtain knowledge of their adolescents’ whereabouts through adolescents’ own sharing or disclosure of information. Early adolescent girls generally seem to share more information with their parents than boys (Kapetanovic et al. 2017), although a vast literature indicates that adolescent disclosure is the strongest predictor of parental knowledge of an adolescent’s activities regardless of adolescent gender (Keijsers et al. 2010a). In addition, studies indicate that adolescent disclosure is an important aspect of the parent–adolescent relationship (Tilton-Weaver 2014) which in turn is associated with less engagement in problem behaviors such as delinquency (Kapetanovic et al. 2019a), substance use (Kapetanovic et al. 2019b), and internalizing (Hamza and Willoughby 2011) and externalizing problems (Racz and McMahon 2011). Importantly, adolescent disclosure demonstrates these unique protective effects even after parents’ own communication strategies are statistically controlled (Kerr et al. 2010). When adolescents share information, parents become more knowledgeable of adolescent whereabouts and could be provided with more opportunities to give guidance and support without being intrusive. As a result, adolescents could be protected from development of psychological problems.

Adolescents can also manage information by withholding it from their parents. As adolescents become more autonomous and independent from their parents, they utilize the secrecy communication strategy to choose what to divulge to their parents and what information to withhold. Adolescents withhold information and keep secrets from parents for several reasons, including avoiding disapproval, gaining a sense of autonomy (Marshall et al. 2005), or protecting their privacy (Rote and Smetana 2016). Particularly boys seem to increase their secrecy toward parents over the course of early to mid-adolescence (Keijsers et al. 2010b). Although secrecy may be a normative process in adolescent development, empirical research consistently shows that secrecy is psychologically disadvantageous to adolescent development (Finkenauer et al. 2002) and is associated with higher levels of internalizing and externalizing behaviors over time (Frijns et al. 2010). It is possible that adolescents who withhold information from parents are less likely to receive guidance and support from their parents and could therefore experience poorer psychosocial development.

### Adolescents Influence Parents

Adolescents cannot be seen as a separate entity from the contexts they live in, but instead must be viewed as active agents who interact with all levels of their developing contextual system (Sameroff 2010). Applied to parent–adolescent communication patterns, this means that parents and adolescents both demonstrate agency in shaping their mutual communication patterns, and actively influence each other (Kuczynski and De Mol 2015). In line with such reasoning, developmental scientists have shown that parents adapt their communication strategies as a result of adolescents’ behavior (Lansford et al. 2018b).

Specifically, during the last decade, many studies have tested unidirectional associations between parent–adolescent communication and adolescent adjustment. However, only a few studies have examined reciprocal associations between parent-driven communication efforts (i.e., parent solicitation and control) and adolescent adjustment (i.e., externalizing and internalizing problems) and between adolescent-driven communication efforts (i.e., adolescent disclosure and secrecy) and adolescent adjustment (i.e., externalizing and internalizing problems). These bodies of work have produced inconsistent results. Some studies that investigate parent communication efforts show that parent communication efforts unidirectionally predict, but are not predicted by, adolescent adjustment (Kerr et al. 2010). However, other studies suggest that parent communication efforts are reciprocally linked with adolescent internalizing and externalizing behaviors such that parental efforts predict adolescent behavior and vice versa (Hamza and Willoughby 2011). Some studies that investigate adolescent communication efforts indicate that adolescent secrecy unidirectionally predicts adolescent internalizing problems (Frijns et al. 2010), whereas in others adolescent disclosure predicts and is predicted by adolescent internalizing problems (Hamza and Willoughby 2011). Studies simultaneously investigating links between parents’ communication efforts and adolescents’ communication efforts are scarce. However there are indications that adolescent secrecy and parent solicitation are positively linked concurrently, yet not longitudinally (Villalobos Solíss et al. 2015). Thus, though reciprocal associations among parent communication efforts, adolescent communication efforts, and adolescent behavioral adjustment have been proposed in theoretical conceptualizations, reciprocal associations among all three constructs have not previously been tested together in one model. Doing so might resolve inconsistencies in the parent communication-adolescent adjustment and adolescent communication-adolescent adjustment literatures by identifying exactly which parent- and adolescent-driven communication patterns are uniquely associated with adolescent adjustment over time.

### Are Links between Parent–Adolescent Communication and Adolescent Development Similar across Cultures?

Parent–adolescent interactions may be influenced by their cultural context (Bornstein et al. 2011). Behavior of individuals or a group of people cannot be separated from the social context in which they live (Sameroff 2010). Accordingly, parents’ values and beliefs are embedded within their specific social context, such as the community or culture. Specifically, some cultures’ norms allow adolescents greater agency in communicating with their parents and shaping parent–adolescent relationships, whereas others emphasize adolescents’ obedience and restrict adolescents’ agency to shape such relationships (Smetana 2017). For example, parents in Jordan and Sweden hold parenting views that encourage adolescent autonomy, but parenting in China and Kenya is characterized by more authoritarian styles that accord adolescents less autonomy (Putnick et al. 2012). The use of specific parenting practices may vary across cultures, but their effect on adolescent adjustment may or may not differ across cultures (Bornstein 2012).

In what ways are parent and adolescent communication efforts linked to adolescent adjustment across cultures? Given differences across cultures in adolescent agency, it is possible that parent–adolescent communication efforts are differentially associated with one another and with adolescent functioning in different cultures. Another possibility is that parent–adolescent communication efforts are universally linked with one another and with adolescent functioning. Current research, however, has not investigated the moderating effects of culture on these associations. To date, research linking adolescent functioning with both parents’ and adolescents’ communication efforts is mostly conducted within European American and European samples (Smetana 2008). One exception included Palestinian refugee youth in Jordan and showed that adolescent disclosure and secrecy were associated with norm breaking behaviors and internalizing problems, but only when mothers were high in behavior control (Ahmad et al. 2015). Another exception, including non-Latino White and Latino adolescents in the United States, showed that adolescent disclosure was negatively associated with adolescent depressive symptoms whereas parent solicitation was positively associated with adolescent depressive symptoms over time and across ethnicity (Fernandez et al. 2018). Although these findings are important first steps, more culturally inclusive research is needed to understand the links between parent–adolescent communication efforts and adolescent adjustment. To fill this knowledge gap, the current study utilizes longitudinal samples of adolescents in 12 cultural groups in nine countries to examine how bidirectional associations among parent-communication, adolescent-communication, and adolescent adjustment vary across culture. Understanding similarities and differences across cultures in bidirectional communication patterns could aid in the identification of culture-specific targets for preventing and intervening to mitigate adolescents’ internalizing and externalizing problems.

## Current Study

The current study examines associations among parental communication efforts (i.e., solicitation and behavior control) and adolescent communication efforts (i.e., adolescent disclosure and secrecy) and adolescent psychological problems (i.e., internalizing and externalizing problems) in a large multicultural sample. Because earlier research suggests that parenting practices differ based on adolescent gender (Leaper and Farkas 2015) and parent socioeconomic status, including education level (Morawska et al. 2009), adolescent gender and parent education level were used as covariates in all models. Based on aforementioned unidirectional studies that demonstrate that parent solicitation and behavioral control are protective (e.g., Dishion and McMahon 1998), the first hypothesis was that parent solicitation and behavioral control would be linked to lower levels of adolescent problems over time. In line with the reconceptualization of parent–adolescent communication patterns that emphasized the influence of adolescent information management (Stattin and Kerr 2000), the second hypothesis was that adolescent disclosure would be linked to lower levels of adolescent problems over time, whereas adolescent secrecy would be linked to higher levels of adolescent psychological problems over time. Based on the tenet of developmental science that emphasizes bidirectional processes in parent–adolescent interactions (Kuczynski and De Mol 2015) and findings in the previous literature (Kapetanovic et al. 2019a), the third hypothesis was that the links among parent–adolescent communication efforts and adolescent psychological problems would be reciprocal so that (a) adolescent psychological problems would be negatively and reciprocally related to parent solicitation, control, and adolescent disclosure and positively to adolescent secrecy, (b) parent solicitation, control, and adolescent disclosure would be positively and reciprocally related, and (c) parent solicitation, behavioral control, and adolescent disclosure would be negatively and reciprocally associated with adolescent secrecy. Cultural moderation in the links between parent–adolescent communication and adolescent psychological problems will also be tested. However, given the paucity of cross-cultural research in the area, no specific hypotheses regarding moderation by culture were made.

## Methods

### Participants

Participants included 1087 adolescents (*M* = 13.19 years, *SD* = 0.90, 50% girls at year 1 (age 13) of two years of data collection (ages 13 and 15)). Families were recruited from 12 distinct ethnic/cultural groups across 9 countries including: Shanghai, China (*n* = 85); Medellín, Colombia (*n* = 85); Naples (*n* = 95) and Rome (*n* = 99), Italy; Zarqa, Jordan (*n* = 104); Kisumu, Kenya (*n* = 90); Manila, Philippines (*n* = 91); Trollhättan/Vänersborg, Sweden (*n* = 83); Chiang Mai, Thailand (*n* = 100); and Durham, NC, United States (*n* = 93 White, *n* = 90 Black, *n* = 72 Latino). Participants were recruited through school letters. Most adolescents had parents who lived together (82%) and were biological parents (97%); nonresidential and non-biological parents also provided data. Sampling included families from each country’s majority ethnic group, except in Kenya where Luo (3rd largest ethnic group, 13% of population) was sampled, and in the U.S., where equal proportions of White, Black, and Latino families were sampled. Socioeconomic status and parental education were sampled in proportions representative of each recruitment area. Adolescent age and gender did not vary across countries. Data for the present study were drawn from interviews during both study years (when adolescents were approximately ages 13 and 15). At age 15, 92% (*n* = 997) of the original sample continued to provide data.

### Procedure

Data used in the current study were pulled from a larger longitudinal investigation that also included adolescents’ mothers and fathers. However, mother and father reports were not included in the present sample because only adolescents reported on parent–child communication patterns. The study was described to potential participants as a longitudinal study examining parenting and child development, and both parents, in addition to the target child, were invited to participate in the study. Participants were recruited through public and private schools (to increase socioeconomic diversity and representativeness of the sample) in all nine countries. Response rates varied across countries (from 24% to nearly 100%), primarily because of differences in the schools’ roles in recruiting. For example, in China, once the schools agreed to participate, the parents agreed to participate as well, and interviews were conducted at the schools, leading to participation rates of nearly 100%. In the United States, after schools agreed to help with recruitment, the interview team was allowed to leave letters explaining the study at the school for teachers to send home with students. If parents were willing to have their family participate, they returned a letter to the school indicating their willingness to participate. The research team then contacted them directly to arrange an interview at a time and place that was convenient for the families. Based on the number of letters that were left at schools for teachers to distribute compared to the number of letters returned by parents, the estimated response rate was 24%. Unfortunately, the estimation of response rates for all sites is not possible because in some cases, there is no record of the number of students who were potentially invited to participate versus those who actually agreed to participate due to the differing ways in which schools informed parents about the study (e.g., paper letters versus email contact or verbal announcement).

Measures were administered in the predominant language of each country, following forward- and back-translation and meetings to resolve any item-by-item ambiguities in linguistic or semantic content (Erkut 2010). Interviews lasted 2 h in participant-chosen locations and were conducted after parent consent and adolescent assent were given. Data collection at each of the site was led by the site principal investigator and coordinated from the local university where the site principal investigator had a faculty appointment. Interviewers at each cultural site were research assistants (e.g., graduate students, local community members) trained by the principal investigators. Participants were given the choice of completing the measures in writing or orally. Families were given modest monetary compensation for participating or compensated in other ways deemed appropriate by local IRBs.

### Measures

#### Demographics

Adolescent biological sex and parent education were included in analyses as covariates. Adolescent biological sex was measured via a binary variable (0 = female, 1 = male) and parent education was calculated as the maximum number of years of education that any parent in the household completed (*M* = 13.80, *SD* = 4.13). Average years of parental education ranged across cultures from 10.89 years (in the US Latino sample) to 17.95 years (in the US White sample). Therefore, overall the sample was high-school educated, with some variation across cultures.

#### Adolescent externalizing and internalizing behavior

Adolescents completed the Youth Self Report Form of the Adolescent Behavior Checklist (Achenbach and Rescorla 2001) to capture their externalizing and internalizing behavior at ages 13 and 15. Adolescents were asked to rate how true each item was during the last six months (0 = *not true*, 1 = *somewhat or sometimes true*, 2 = *very or often true*). The *Externalizing Behavior* scale summed across 30 items and captured behaviors such as lying, vandalism, bullying, substance use, disobedience, and physical violence. The *Internalizing Behavior* scale summed across 29 items and measured behaviors and emotions such as loneliness, self-consciousness, nervousness, sadness, and anxiety. The Achenbach measures are among the most widely used instruments in international research, with translations in over 100 languages and strong, well-documented psychometric properties (e.g., Achenbach and Rescorla 2001). This measure demonstrated adequate reliability across cultures at age 13 (Internalizing α = 0.89; Externalizing α = 0.87) and age 15 (Internalizing α = 0.73; Externalizing α = 0.75) and has demonstrated reliability and validity within all cultural groups in the present sample in past work (Lansford et al. 2018a). Moreover, measurement invariance and consistency of the Youth Self Report factor structure has been demonstrated in numerous cultural groups worldwide, including those examined in the current study (e.g., Yarnell et al. 2013). Higher scores indicated more problematic externalizing/internalizing behavior.

#### Parent–adolescent communication: adolescent disclosure, adolescent secrecy, parent behavioral control, parent solicitation

Parent–adolescent communication patterns were assessed at ages 13 and 15 by adolescent reports of adolescent disclosure, adolescent secrecy, parent behavior control, and parent solicitation on items from the Youth Knowledge, Disclosure, Control, and Solicitation Scale (Stattin and Kerr 2000; see Supplementary Table 1 for a full list of items). Adolescents were asked how frequently they engaged in disclosure and secrecy, and how often their parents engaged in control and solicitation, on a 0 = *Never* to 3 = *Always* scale. Adolescent disclosure was measured by summing three items assessing how often adolescents share information with their parents (e.g., “Do you like to tell your parents where you went and what you did during the evening?”). Adolescent secrecy was measured by summing two items assessing how often adolescents kept secrets from their parents (e.g., “Do you hide a lot from your parents about what you do during nights and weekends?”). Parent solicitation was measured by summing four items assessing how often parents initiate conversation with their adolescent (e.g., “How often do your parents ask you what happened during your free time?”). Parent behavioral control was measured by summing six items assessing how often parents attempted to remain aware, establish clear expectations for, and set limits on adolescent behavior (e.g., “Do your parents require that you tell them how you spend your money?). The Youth Knowledge, Disclosure, Control, and Solicitation Scale has adequate reliability, convergent and discriminant validity, and measurement invariance across a variety of cultures including many of those examined in the current study (e.g., Lionetti et al. 2016; Frijns et al. 2010). Moreover, adolescent disclosure (Age 13 α = 0.72; Age 15 α = 0.72), adolescent secrecy (Age 13 α = 0.63; Age 15 α = 0.69), parent behavioral control (Age 13 α = 0.79; Age 15 α = 0.82), and parent solicitation (Age 13 α = 0.75; Age 15 α = 0.78) each demonstrated reliability in the current study. Higher scores indicate greater adolescent disclosure, adolescent secrecy, parent behavioral control, and parent solicitation.

### Analytic Plan

Mplus Version 8 (Muthén and Muthén 1998) was used to evaluate study hypotheses. In the path model, stability paths for each variable from age 13 to age 15 were included (e.g., age-13 adolescent externalizing behavior predicted age-15 adolescent externalizing behavior) and contemporaneous measures were correlated at both study time points (e.g., age-13 adolescent disclosure and age-13 adolescent secrecy were correlated). Additionally, paths from each age-13 measure were fit predicting each age-15 measure. So, for example, paths from age-13 adolescent externalizing behavior, internalizing behavior, disclosure, and secrecy, and from parent behavioral control and solicitation, as well as from adolescent gender and parent education predicted all age-15 variables. Once paths were fit, multiple-group comparisons of the 12 cultural groups were conducted to examine cultural differences. In accordance with prior work (Rothenberg et al. 2019; Muthén and Muthén 1998), all paths were initially constrained to be equal across cultures. Then, paths were iteratively freed to vary across cultures if a χ^2^ difference test revealed that the model fit was significantly better when the path was freed. Paths were freed to vary and tested using χ^2^ difference tests in the same order in every model. Analyzing the data in this way allowed precise identification of the effects that vary across cultural groups.

## Results

The means and standard deviations of all study variables in each culture are shown in Table 1.

**Table 1:** Descriptive statistics for all study variables

	Cultural group											
	China (*M*, SD) or % Male	Naples, Italy (*M*, SD) or % Male	Rome, Italy (*M*, SD) or % Male	Kenya (*M*, SD) or % Male	Philippines (*M*, SD) or % Male	Thailand (*M*, SD) or % Male	Sweden (*M*, SD) or % Male	USB(*M*, SD) or % Male	USW (*M*, SD) or % Male	USL (*M*, SD) or % Male	Colombia (*M*, SD) or % Male	Jordan (*M*, SD) or % Male
**Age 13 Variables**												
Age 13 adolescent communication patterns												
Disclosure	5.55 (2.41)	5.32 (2.34)	4.99 (2.45)	6.83 (2.15)	5.85 (2.12)	5.44 (2.35)	6.02 (2.02)	5.55 (2.45)	5.19 (2.08)	5.85 (2.05)	4.88 (2.36)	6.77 (2.43)
Secrecy	1.33 (1.29)	1.10 (1.48)	1.12 (1.36)	1.19 (1.59)	1.67 (0.149)	2.26 (1.61)	0.61 (0.81)	1.29 (1.65)	1.07 (1.30)	0.99 (1.19)	0.96 (1.28)	2.02 (1.91)
Age 13 parent communication patterns												
Control	11.12 (4.83)	13.74 (3.47)	13.34 (4.02)	13.88 (3.72)	13.51 (3.58)	13.4 (4.38)	12.54 (3.92)	13.28 (3.65)	13.74 (3.23)	14.61 (3.04)	15.52 (3.11)	14.83 (3.28)
Solicitation	6.62 (2.57)	6.52 (2.55)	6.14 (2.46)	8.06 (2.62)	7.01 (2.69)	7.16 (2.68)	7.04 (2.48)	6.64 (2.80)	7.27 (2.61)	6.67 (2.74)	6.25 (3.03)	8.73 (3.48)
Age 13 adolescent psychological problems												
Externalizing behavior	5.68 (4.97)	9.70 (5.85)	10.99 (6.74)	7.87 (7.01)	12.41 (6.67)	11.71 (7.99)	9.18 (4.95)	10.11 (8.65)	10.66 (6.22)	8.26 (6.45)	10.46 (6.97)	13.72 (8.55)
Internalizing behavior	7.61 (7.11)	13.42 (8.41)	13.37 (7.83)	13.70 (8.86)	18.57 (9.10)	14.77 (8.33)	8.06 (6.31)	12.12 (8.97)	13.29 (9.52)	9.07 (6.84)	14.22 (8.65)	13.84 (7.79)
Demographic covariates												
Adolescent gender	50%	48%	53%	40%	51%	51%	51%	48%	58%	47%	44%	53%
Years of parent education	14.46 (2.82)	11.38 (4.36)	14.90 (4.21)	12.77 (3.46)	14.80 (3.97)	13.22 (4.41)	14.61 (2.65)	14.12 (2.50)	17.95 (3.05)	10.89 (3.86)	11.70 (5.51)	14.21 (2.47)
**Age 15 Variables**												
Age 15 adolescent communication patterns												
Disclosure	5.45 (2.36)	5.32 (2.29)	4.77 (2.26)	6.35 (2.25)	5.71 (2.02)	5.15 (2.51)	5.58 (2.04)	5.67 (2.46)	4.70 (2.16)	6.03 (2.07)	4.89 (2.38)	6.47 (2.67)
Secrecy	1.58 (1.16)	1.01 (1.27)	1.27 (1.46)	1.10 (1.35)	1.69 (1.21)	1.93 (1.39)	1.12 (1.27)	1.15 (1.39)	1.47 (1.51)	1.02 (1.34)	1.28 (1.39)	2.30 (2.24)
Age 15 parent communication patterns												
Control	12.68 (4.38)	12.79 (3.98)	12.25 (4.07)	13.92 (3.50)	12.91 (3.82)	13.21 (4.15)	10.92 (4.38)	12.75 (4.11)	12.38 (3.85)	14.08 (3.38)	13.65 (3.40)	13.91 (4.29)
Solicitation	6.62 (2.57)	6.52 (2.55)	6.14 (2.46)	8.06 (2.62)	7.01 (2.69)	7.16 (2.68)	7.05 (2.48)	6.64 (2.80)	7.27 (2.61)	6.66 (2.74)	6.25 (3.03)	8.73 (3.48)
Age 15 adolescent psychological problems												
Externalizing behavior	5.77 (4.30)	11.62 (6.39)	12.47 (7.01)	6.83 (4.81)	13.24 (6.85)	13.51 (7.78)	10.66 (5.88)	9.34 (7.91)	12.09 (7.61)	8.97 (6.73)	13.37 (8.56)	13.56 (8.19)
Internalizing behavior	10.54 (7.26)	15.98 (9.83)	15.12 (8.92)	13.92 (6.81)	18.29 (8.39)	15.94 (9.17)	11.25 (8.01)	12.24 (10.04)	16.25 (11.21)	11.64 (8.33)	17.69 (11.78)	12.22 (8.71)

*USB* US Black, *USW* US White, *USL* US Latino

### Missing Data

At age 15, 92% (*n* = 997) of the original sample continued to provide data. Attrited participants did not significantly differ from the original sample on parent education, adolescent externalizing behavior, disclosure, secrecy, or parent solicitation at age 13. However, attrited participants were more likely to be boys (*M*_*Attrited*_ = 54% boys, *M*_*Retained*_ = 48% boys, *t*(1334) = −2.05, *p* = 0.04) and reported lower levels of internalizing behavior (*M*_*Attrited*_ = 11.26, *M*_*Retained*_ = 13.15, *t*(1085) = 2.45, *p* = 0.01) and parental control (*M*_*Attrited*_ = 12.22, *M*_*Retained*_ = 13.76, *t*(1082) = 4.53, *p* = 0.01) at age 13. Therefore, all age-13 variables were included in all analyses, and full-information maximum likelihood estimation procedures were utilized in all path analyses to account for missing data (Enders 2010).

### Fitting Appropriate Path Analytic Model

**Fig. 1:**
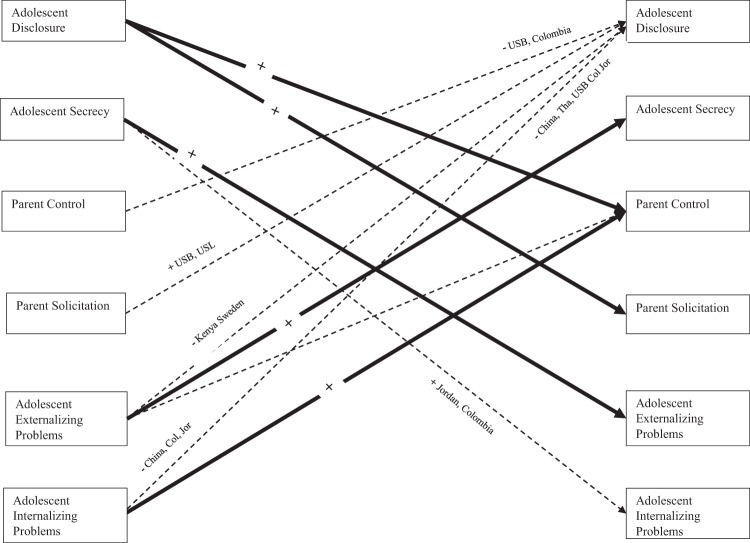
Depiction of statistically significant paths in final analytic model. + Indicates positive association. − Indicates negative association. Solid lines indicate paths that are positive and significant in all cultures. Dashed lines indicate paths that are significant in some cultures but not others. For these dashed paths, cultures where the path is significant are noted. Col = Colombia; Jor = Jordan; Tha = Thailand; USB = United States Black; USL = US Latino. Nonsignificant paths and demographic covariates are not depicted due to space constraints. Additionally, autoregressive paths are not depicted despite being significant in some or all cultures due to space constraints. For a full report of all parameter estimates for all paths, refer to Table 2

Skewness and kurtosis estimates for all continuous variables estimated in the models fell in acceptable ranges (skew <2.0, kurtosis < 7.0). Model fit evaluation was based on recommended fit index cut-off values that indicate excellent fit (CFI/TLI > 0.95, RMSEA < 0.05, SRMR < 0.08; Kline 2011). Standardized parameter estimates and standard errors are provided in Table 2, and substantive results from this model are depicted in Fig. 1.

**Table 2:** Results from path analyses predicting age 15 outcomes from age 13 predictors, adolescent gender, and parent education

Cultural group												
	China (β, SE)	Naples, Italy (β, SE)	Rome, Italy (β, SE)	Kenya (β, SE)	Philippines (β, SE)	Thailand (β, SE)	Sweden (β, SE)	USB (β, SE)	USW (β, SE)	USL (β, SE)	Colombia (β, SE)	Jordan (β, SE)
**Age 13 predictors of age 15 child disclosure**												
Age 13 adolescent communication patterns												
Disclosure	**0**.26 (0.05)*	**0**.**29 (0**.05)*	**0**.**31 (0**.05)*	**0**.**25 (0**.04)*	**0**.**27 (0**.04)*	**0**.**24 (0**.04)*	**0**.**28 (0**.05)*	**0**.**27 (0**.04)*	**0**.**25 (0**.04)*	**0**.**28 (0**.05)*	**0**.**27 (0**.05)*	**0**.**24 (0**.04)*
Secrecy	0.00 (0.03)	0.00 (0.04)	0.00 (0.03)	0.00 (0.04)	0.00 (0.04)	0.00 (0.03)	0.00 (0.02)	0.00 (0.04)	0.00 (0.03)	0.00 (0.03)	0.00 (0.03)	0.00 (0.04)
Age 13 parent communication patterns												
Control	−0.12 (0.17)	−0.04 (0.11)	0.08 (0.09)	0.01 (0.12)	0.07 (0.10)	0.01 (0.09)	−0.07 (0.12)	−**0**.25 (0.09)*	−0.01 (0.10)	−0.12 (0.14)	−**0.22 (0**.10)*	0.14 (0.10)
Solicitation	0.12 (0.15)	0.01 (0.11)	0.14 (0.09)	−0.12 (0.10)	0.12 (0.10)	0.11 (0.10)	0.18 (0.10)	0.25 (0.10)*	0.07 (0.10)	0.31 (0.12)*	0.09 (0.10)	−0.08 (0.09)
Age 13 adolescent psychological problems												
Externalizing behavior	0.27 (0.26)	0.09 (0.12)	−0.13 (0.11)	−0.27 (0.12)*	0.20 (0.11)	−0.20 (0.12)	−0.41 (0.10)*	−0.05 (0.11)	−0.04 (0.13)	−0.08 (0.15)	0.09 (0.11)	−0.01 (0.11)
Internalizing behavior	−0.59 (0.20)*	−0.09 (0.12)	−0.10 (0.11)	0.18 (0.12)	−0.04 (0.11)	0.05 (0.11)	0.16 (0.11)	0.15 (0.10)	−0.14 (0.13)	−0.10 (0.14)	−0.26 (0.10)*	−0.20 (0.10)*
Demographic covariates												
Adolescent male gender	−0.04 (0.14)	−0.03 (0.10)	−0.14 (0.08)	−0.02 (0.09)	−0.17 (0.08)*	−**0.16 (0.09)**^**+**^	−0.01 (0.09)	−0.09 (0.08)	−0.29 (0.09)	−0.27 (0.11)*	−0.11 (0.09)	0.34 (0.09)*
Years of parent education	−0.01 (0.02)	−0.01 (0.04)	−0.01 (0.04)	−0.01 (0.03)	−0.01 (0.04)	−0.01 (0.03)	−0.01 (0.02	−0.01 (0.02)	−0.01 (0.03)	−0.01 (0.04)	−0.01 (0.04)	0.00 (0.02)
**Age 13 predictors of age 15 child secrecy**												
Age 13 adolescent communication patterns												
Disclosure	−0.04 (0.05)	−0.04 (0.04)	−0.03 (0.04)	−0.03 (0.04)	−0.03 (0.04)	−0.03 (0.04)	−0.03 (0.04)	−0.03 (0.04)	−0.03 (0.03)	−0.03 (0.04)	−0.03 (0.04)	−0.02 (0.02)
Secrecy	−0.08 (0.15)	0.38 (0.08)*	0.24 (0.09)*	0.08 (0.12)	0.24 (0.10)*	0.12 (0.10)	0.20 (0.10)*	−0.03 (0.11)	0.29 (0.08)*	0.09 (0.14)	0.39 (0.10)*	0.16 (0.09)
Age 13 parent communication patterns												
Control	−0.02 (0.06)	−0.01 (0.04)	−0.01 (0.04)	−0.01 (0.04)	−0.01 (0.04)	−0.01 (0.04)	−0.01 (0.04)	−0.01 (0.04)	−0.01 (0.03)	−0.01 (0.03)	−0.01 (0.03)	−0.01 (0.02)
Solicitation	0.00 (0.04)	0.01 (0.04)	0.00 (0.03)	0.00 (0.04)	0.01 (0.04)	0.00 (0.04)	0.01 (0.04)	0.01 (0.04)	0.00 (0.03)	0.01 (0.04)	0.01 (0.04)	0.00 (0.03)
Age 13 adolescent psychological problems												
Externalizing behavior	0.15 (0.04)*	0.17 (0.04)*	0.17 (0.04)*	0.18 (0.05)*	0.19 (0.05)*	0.20 (0.05)*	0.14 (0.04)*	0.22 (0.05)*	0.14 (0.04)*	0.18 (0.05)*	0.18 (0.05)*	0.13 (0.03)*
Internalizing behavior	0.00 (0.04)	0.00 (0.05)	0.00 (0.04)	0.00 (0.04)	0.00 (0.05)	0.00 (0.04)	0.00 (0.03)	0.00 (0.04)	0.00 (0.04)	0.00 (0.03)	0.00 (0.04)	0.00 (0.02)
Demographic covariates												
Adolescent Male Gender	0.09 (0.04)*	0.08 (0.04)*	0.07 (0.03)*	0.08 (0.03)*	0.09 (0.04)*	0.08 (0.03)*	0.08 (0.03)*	0.07 (0.03)*	0.07 (0.03)*	0.08 (0.03)*	0.08 (0.03)*	0.05 (0.02)*
Years of parent education	−0.04 (0.18)	−0.03 (0.08)	0.26 (0.09)*	0.01 (0.11)	0.11 (0.11)	−0.13 (0.11)	−**0.25 (0**.10)*	−0.13 (0.10)	−0.11 (0.08)	−0.09 (0.14)	−0.11 (0.10)	0.04 (0.09)
**Age 13 predictors of age 15 parent control**												
Age 13 adolescent communication patterns												
Disclosure	**0.06 (0.03)**^**+**^	**0.07 (0.04)**^**+**^	**0.07 (0.04)**^**+**^	**0.07 (0.04)**^**+**^	**0.06 (0.03)+**	**0.06 (0.03)**^**+**^	**0.06 (0.03)**^**+**^	**0.07 (0.04)**^**+**^	**0.06 (0.03)**^**+**^	**0.08 (0.04)**^**+**^	**0.08 (0.04)**^**+**^	**0.07 (0.03)**^**+**^
Secrecy	−0.04 (0.03)	−0.06 (0.03)	−0.05 (0.03)	−0.07 (0.04)	−0.06 (0.04)	−0.06 (0.03)	−0.03 (0.02)	−0.06 (0.04)	−0.05 (0.03)	−0.06 (0.04)	−0.05 (0.03)	−0.07 (0.04)
Age 13 parent communication patterns												
Control	0.04 (0.16)	0.46 (0.07)*	0.33 (0.09)*	0.33 (0.11)*	0.12 (0.10)	0.45 (0.08)*	0.39 (0.10)*	0.10 (0.08)	0.56 (0.07)*	0.11 (0.13)	0.24 (0.09)*	0.04 (0.11)
Solicitation	0.05 (0.03)	0.06 (0.03)	0.05 (0.03)	0.06 (0.04)	0.06 (0.04)	0.05 (0.03)	0.05 (0.03)	0.06 (0.03)	0.05 (0.03)	0.08 (0.05)	0.08 (0.05)	0.07 (0.04)
Age 13 adolescent psychological problems												
Externalizing behavior	−0.38 (0.14)*	0.10 (0.08)	−0.05 (0.10)	−0.12 (0.11)	0.17 (0.10)	−**0.16 (0.09)**^**+**^	0.18 (0.10)	−0.38 (0.08)*	−0.15 (0.09)	−0.23 (0.13)	−0.21 (0.11)*	−**0.20 (0.10)**^**+**^
Internalizing behavior	0.07 (0.03)*	0.10 (0.04)*	0.10 (0.04)*	0.12 (0.05)*	0.11 (0.04)*	0.09 (0.01)*	0.07 (0.03)*	0.10 (0.04)*	0.11 (0.04)*	0.10 (0.04)*	0.12 (0.05)*	0.08 (0.03)*
Demographic covariates												
Adolescent Male Gender	−0.31 (0.13)	−0.13 (0.08)	−0.16 (0.09)	−0.02 (0.09)	0.02 (0.09)	−0.06 (0.09)	0.14 (0.09)	−0.07 (0.08)	−0.12 (0.08)	−0.16 (0.11)	−0.25 (0.09)*	0.20 (0.10)*
Years of parent education	−0.20 (0.16)	−0.08 (0.08)	−0.04 (0.09)	0.14 (0.09)	0.13 (0.10)	0.15 (0.08)	0.13 (0.10)	−0.11 (0.07)	−0.15 (0.08)	0.24 (0.12)*	0.05 (0.10)	0.04 (0.08)
**Age 13 predictors of age 15 parent solicitation**												
Age 13 adolescent communication patterns												
Disclosure	**0.08 (0.05)**^**+**^	0.07 (0.04)*	0.09 (0.04)*	0.07 (0.04)*	0.07 (0.03)*	0.07 (0.03)*	**0.07 (0.04)+**	0.06 (0.03)*	0.07 (0.04)*	0.07 (0.04)*	0.07 (0.04)*	0.08 (0.04)*
Secrecy	−0.03 (0.04)	−0.03 (0.04)	−0.03 (0.04)	−0.03 (0.04)	−0.03 (0.04)	−0.03 (0.04)	−0.02 (0.02)	−0.03 (0.03)	−0.03 (0.03)	−0.02 (0.03)	−0.02 (0.03)	−0.04 (0.05)
Age 13 parent communication patterns												
Control	−0.02 (0.05)	−0.01 (0.03)	−0.01 (0.04)	−0.01 (0.04)	−0.01 (0.03)	−0.01 (0.04)	−0.01 (0.04)	−0.01 (0.03)	−0.01 (0.03)	−0.01 (0.03)	−0.01 (0.03)	−0.01 (0.03)
Solicitation	0.21 (0.14)	0.46 (0.07)*	0.41 (0.08)*	0.03 (0.10)	0.38 (0.08)*	0.29 (0.09)*	**0**.**48 (0**.08)*	0.21 (0.10)*	0.42 (0.08)*	0.52 (0.09)*	0.26 (0.10)*	0.13 (0.11)
Age 13 adolescent psychological problems												
Externalizing behavior	−0.02 (0.03)	−0.02 (0.04)	−0.03 (0.05)	−0.03 (0.04)	−0.03 (0.04)	−0.03 (0.04)	−0.03 (0.04)	−0.03 (0.04)	−0.03 (0.04)	−0.03 (0.04)	−0.03 (0.04)	−0.04 (0.05)
Internalizing behavior	−0.03 (0.04)	−0.03 (0.04)	−0.04 (0.04)	−0.03 (0.04)	−0.03 (0.04)	−0.03 (0.04)	−0.03 (0.03)	−0.03 (0.04)	−0.04 (0.05)	−0.03 (03)	−0.03 (0.04)	−0.03 (0.04)
Demographic covariates												
Adolescent Male Gender	−0.06 (0.04)	−0.05 (0.03)	−0.06 (0.04)	−0.06 (0.03)	−0.05 (0.03)	−0.05 (0.03)	−0.06 (0.03)	−0.04 (0.03)	−0.06 (0.03)	−0.06 (0.03)	−0.05 (0.03)	−0.05 (0.03)
Years of parent education	−0.03 (0.03)	−0.04 (0.04)	−0.05 (0.04)	−0.03 (0.03)	−0.04 (0.03)	−0.04 (0.03)	−0.03 (0.02)	−0.02 (0.02)	−0.03 (0.03)	−0.04 (0.03)	−0.05 (0.04)	−0.02 (0.02)
**Age 13 predictors of age 15 externalizing behavior**												
Age 13 adolescent communication patterns												
Disclosure	−0.04 (0.05)	−0.03 (0.03)	−0.03 (0.03)	−0.03 (0.04)	−0.03 (0.03)	−0.02 (0.03)	−0.03 (0.03)	−0.02 (0.03)	−0.02 (0.03)	−0.02 (0.03)	−0.02 (0.03)	−0.02 (0.03)
Secrecy	0.11 (0.04)*	0.08 (0.03)*	0.07 (0.03)*	0.12 (0.05)*	0.08 (0.03)*	0.07 (0.03)*	0.05 (0.02)*	0.07 (0.03)*	0.06 (0.02)*	0.06 (0.02)*	0.05 (0.02)*	0.08 (0.03)*
Age 13 parent communication patterns												
Control	−0.07 (0.06)	−0.03 (0.03)	−0.03 (0.03)	−0.05 (0.04)	−0.03 (0.03)	−0.03 (0.03)	−0.04 (0.03)	−0.03 (0.02)	−0.03 (0.02)	−0.03 (0.02)	−0.02 (0.02)	−0.02 (0.02)
Solicitation	0.02 (0.04)	0.02 (0.03)	0.01 (0.03)	0.02 (0.04)	0.02 (0.03)	0.01 (0.03)	0.02 (0.03)	0.01 (0.03)	0.01 (0.03)	0.02 (0.03)	0.01 (0.03)	0.02 (0.03)
Age 13 adolescent psychological problems												
Externalizing behavior	0.39 (0.13)*	0.48 (0.07)*	0.70 (0.05)*	**0.17 (0.09)**^**+**^	0.47 (0.08)*	0.62 (0.06)*	0.65 (0.06)*	0.47 (0.07)*	0.59 (0.06)*	0.67 (0.07)*	0.61 (0.06)*	0.59 (0.06)*
Internalizing behavior	0.04 (0.05)	0.03 (0.04)	0.03 (0.03)	0.05 (0.05)	0.04 (0.04)	0.03 (0.03)	0.03 (0.03)	0.03 (0.03)	0.03 (0.04)	0.03 (0.03)	0.03 (0.03)	0.02 (0.03)
Demographic covariates												
Adolescent Male Gender	−0.10 (0.04)*	−0.06 (0.03)*	−0.06 (0.03)*	−0.08 (0.04)*	−0.06 (0.03)*	−0.05 (0.02)*	−0.07 (0.03)*	−0.05 (0.02)*	−0.05 (0.02)*	−0.06 (0.03)*	−0.05 (0.02)*	−0.05 (0.02)*
Years of parent education	−0.02 (0.03)	0.00 (0.03)	−0.02 (0.03)	0.00 (0.03)	0.00 (0.03)	0.00 (0.03)	0.00 (0.02)	0.00 (0.01)	0.00 (0.02)	0.00 (0.03)	0.00 (0.03)	0.00 (0.01)
**Age 13 predictors of age 15 internalizing behavior**												
Age 13 adolescent communication patterns												
Disclosure	−0.06 (0.04)	−0.04 (0.03)	−0.05 (0.04)	−0.06 (0.04)	−0.04 (0.03)	−0.05 (0.03)	−0.05 (0.04)	−0.04 (0.03)	−0.03 (0.03)	−0.04 (0.03)	−0.04 (0.03)	−0.05 (0.04)
Secrecy	−0.01 (0.10)	−0.06 (0.08)	0.06 (0.07)	0.08 (0.10)	−0.07 (0.08)	0.04 (0.08)	0.10 (0.10)	−0.03 (0.09)	−0.07 (0.08)	0.11 (0.10)	0.28 (0.07)*	0.20 (0.07)*
Age 13 adolescent communication patterns												
Control	0.05 (0.05)	0.03 (0.03)	0.04 (0.03)	0.04 (0.04)	0.03 (0.03)	0.04 (0.04)	0.04 (0.04)	0.03 (0.03)	0.02 (0.02)	0.03 (0.03)	0.02 (0.02)	0.03 (0.03)
Solicitation	0.05 (0.04)	−0.03 (0.03)	0.04 (0.03)	0.05 (0.04)	0.04 (0.03)	0.04 (0.03)	0.04 (0.03)	0.04 (0.03)	0.03 (0.03)	0.04 (0.03)	0.04 (0.03)	0.05 (0.04)
Age 13 adolescent psychological problems												
Externalizing behavior	−0.01 (0.03)	−0.01 (0.03)	−0.01 (0.04)	−0.02 (0.05)	−0.01 (0.04)	−0.01 (0.04)	−0.01 (0.03)	−0.02 (0.04)	−0.01 (0.03)	−0.01 (0.04)	−0.01 (0.03)	−0.02 (0.05)
Internalizing behavior	0.35 (0.13)*	0.57 (0.07)*	0.58 (0.07)*	0.19 (0.10)*	0.65 (0.07)*	0.57 (0.07)*	0.49 (0.09)*	0.46 (0.09)*	0.63 (0.08)*	0.61 (0.08)*	0.45 (0.07)*	0.39 (0.08)*
Demographic covariates												
Adolescent Male Gender	−0.16 (0.10)	−0.24 (0.07)*	−0.18 (0.08)*	−0.11 (0.08)	−0.09 (0.08)	−0.01 (0.08)	**−0.17 (0.09)**^**+**^	−0.24 (0.08)*	−0.27 (0.08)*	−0.26 (0.08)*	−0.33 (0.07)*	−0.18 (0.07)*
Years of parent education	0.00 (0.02)	0.04 (0.03)	0.00 (0.03)	0.00 (0.03)	0.00 (0.03)	0.00 (0.03)	0.00 (0.02)	0.00 (0.02)	0.00 (0.02)	0.00 (0.03)	0.00 (0.03)	0.00 (0.02)

All parameter estimates are standardized. Bold indicates statistically significant parameter estimates

*USA* US Black, *USW* US White, *USL* US Latino

*p ≤ 0.05

^+^*p* ≤ 0.06

In the final model, all contemporaneous correlations among age 13 variables and age 15 variables were freed to vary across cultures, because freeing such variables significantly improved model fit. Additionally, 5 of the 6 stability paths from age 13 to age 15 outcomes were freed to vary across cultures, with the lone exception being the path from age 13 adolescent disclosure to age 15 adolescent disclosure because model fit did not significantly improve when this path was allowed to freely vary across cultures. In contrast, most predictor paths were constrained to be equal across cultures. Specifically, all 5 predictor paths by which age 13 measures predicted age 15 adolescent externalizing behavior, all 5 paths predicting age 15 adolescent secrecy, and all 5 paths predicting age 15 parent solicitation were constrained to be equal across culture. Moreover, 4 of 5 paths predicting age 15 internalizing behavior (with the lone exception being the path from age 13 adolescent secrecy to age 15 adolescent internalizing behavior) and 4 of 5 paths predicting parental control (with the lone exception being the path from age 13 externalizing behavior to age 15 parental control) were constrained to be equal across cultures. The notable exception to this trend was age 15 adolescent disclosure: only 1 of 5 predictor paths (the path from age 13 adolescent secrecy to age 15 adolescent disclosure) was constrained to be equal across cultures. This final model that freed the aforementioned paths and correlations to vary across cultures fit the data better than the initial model that was constrained to be equal across cultures (χ^2^ [627] = 1196.54, *p* < 0.01). Moreover, omnibus measures of model fit indicated that this final model fit the data well (χ^2^ [374] = 421.51, *p* = 0.045, RMSEA = 0.03, CFI = 0.98, TLI = 0.96, SRMR = 0.07), whereas the initial model that constrained all paths to be equal across cultures was a poor fit to the data (χ^2^ [1001] = 1618.04, *p* < 0.01, RMSEA = 0.07, CFI = 0.77, TLI = 0.79, SRMR = .14)

### Path Analysis Results

Complete results for all stability and predictor paths are reported in Table 2. Contemporaneous correlations among study variables were not the focus of the present study because numerous cross-sectional investigations in existing literature already report such contemporaneous findings, and so they are not reported here. Doing so would require the reporting of a total of 516 correlations (43 per cultural group) which was not possible given current space limitations. Correlations are available from the second author on request. Significant covariate effects are reported in Table 2 but not reported further. Additionally, stability paths were included in all study analyses to ensure that significant hypothesized associations emerged even after accounting for stability in behaviors. However, these paths themselves were not a focus of the current study, and therefore will be briefly summarized first here (and are reported in Table 2).

Regarding parent communication variables, higher age-13 parent solicitation predicted higher age-15 parent solicitation in 9 of 12 cultures (all but China, Kenya and Jordan), and higher age-13 parent control predicted higher age-15 parent control in seven of 12 cultures (Naples, Italy; Rome, Italy; Kenya; Thailand; Sweden; U.S. Whites; and Colombia). Regarding adolescent communication variables, greater age-13 adolescent disclosure predicted greater age-15 adolescent disclosure in all cultures, and higher age-13 adolescent secrecy predicted higher age-15 adolescent secrecy in 6 of 12 cultures (Naples, Italy; Rome, Italy; Philippines; Sweden; U.S. White; Colombia). Regarding adolescent psychological problems, greater age-13 internalizing behavior predicted greater age-15 internalizing behavior in all cultures, and greater age-13 externalizing behavior predicted greater age-15 externalizing behavior in all cultures except for Kenya (where *p* = 0.06). The study hypotheses are evaluated after accounting for such behavioral stabilities.

### Hypothesis 1: Parent Communication Predicts Adolescent Psychological Problems

Hypothesis 1 was not supported. Parent solicitation and control did not predict adolescent externalizing or internalizing problems in any culture after accounting for prior levels of externalizing and internalizing problems and adolescent communication efforts.

### Hypothesis 2: Adolescent Communication Predicts Adolescent Psychological Problems

Hypothesis 2 was partially supported. As hypothesized, adolescent secrecy did prospectively predict higher levels of psychological problems over time, but the universality of these associations differed depending on the psychological problem examined. Specifically, higher age-13 adolescent secrecy predicted greater age-15 adolescent externalizing problems in all cultures (Table 2; Fig. 1). However, age-13 secrecy predicted greater age-15 internalizing problems in only two cultures (Colombia and Jordan; Table 2; Fig. 1). Moreover, contrary to the hypothesis adolescent disclosure was not a predictor of either externalizing or internalizing problems in any culture.

### Hypothesis 3: Reciprocity among Parent–Adolescent Communication and Adolescent Psychological Problems

Hypothesis 3 included three sub-hypotheses. Hypothesis 3A was that adolescent psychological problems would be negatively and reciprocally related to parent solicitation, control, and adolescent disclosure, and positively related to adolescent secrecy. Adolescent secrecy was positively and reciprocally associated with adolescent externalizing behavior across cultures, but no other bidirectional links between parent/child communication efforts and adolescent psychological problems were found. Specifically, higher adolescent secrecy at age 13 predicted greater age-15 adolescent externalizing behavior across all cultures, and greater age-13 adolescent externalizing problems also predicted higher age-15 adolescent secrecy in all cultures (Table 2; Fig. 1).

Notably, several additional cross-cultural and culture-specific associations between adolescent psychological problems, parent control, and adolescent disclosure emerged in hypothesized directions, but these associations were unidirectional, as opposed to reciprocal, in nature. Each of these unidirectional associations was characterized by adolescent psychological problems prospectively predicting parent or adolescent communication patterns. Specifically, in all cultures, greater internalizing behavior at age 13 predicted greater parent control at age 15 (Table 2; Fig. 1). Additionally, in Kenya and Sweden, greater externalizing problems at age 13 predicted less adolescent disclosure at age 15, whereas in China, Colombia, and Sweden, greater internalizing problems at age 13 predicted less adolescent disclosure at age 15. Finally, in the China, US Black, Thailand, and Colombian samples, greater externalizing problems at age 13 predicted less parent control at age 15.

Hypothesis 3B was that parent solicitation, parent control, and adolescent disclosure would be positively and reciprocally related. These associations only emerged in specific cultures. Specifically, in the US Black and US Latino samples, greater age-13 parent solicitation led to greater age-15 adolescent disclosure, and greater age-13 adolescent disclosure predicted greater age-15 parent solicitation (Table 2; Fig. 1). Additionally, in the US Black and Colombian samples, greater age-13 adolescent disclosure predicted greater age-15 parent behavior control, but greater age-13 parent behavior control predicted less age-15 adolescent disclosure. So, within these two cultures, these bidirectional processes were reciprocal, but opposite in direction. Interestingly, positive associations between adolescent disclosure and parent solicitation, and adolescent disclosure and parent control did emerge as hypothesized across cultures. However, these associations were unidirectional, instead of reciprocal, in nature. Specifically, first, in all cultures, greater adolescent disclosure at age 13 predicted higher parent solicitation at age 15 (Table 2; Fig. 1). Second, in all cultures, greater adolescent disclosure at age 13 was a nearly significant predictor (*p* = 0.053–0.059) of greater parent control at age 15 (Table 2; Fig. 1).

Hypothesis 3C was not supported. There was no evidence that indicated parent solicitation, control, and disclosure were negatively and reciprocally related to adolescent secrecy.

### Sensitivity Analyses Examining the Moderating Effect of Adolescent Gender

Though no a priori hypotheses about differences in study findings based on adolescent gender were made, exploratory sensitivity analyses were run to examine adolescent gender as a moderator of links among the parent and adolescent communication, and adolescent psychological problems variables so that future investigations could build upon this work. The results of these sensitivity analyses largely indicated that adolescent gender did not appear to play a moderating role in the bidirectional links between parent communication variables and adolescent communication variables, nor between parent and adolescent communication variables and adolescent externalizing or internalizing problems. Moreover, the moderating role of adolescent gender did not substantively vary across cultures. Specifically, out of 360 possible predictive paths that could be moderated by adolescent gender across cultures, only 12 such paths (3.33%) were significantly moderated by gender. Moreover, there did not appear to be any noticeable pattern in significant findings. No culture accounted for more than 3 significant moderating effects, and no more than 4 cultures ever significantly differed along any gender moderated path. Given that the number of moderating paths fell well within the 5% of paths expected to be significant due purely to chance at the *p* < 0.05 threshold, and that there was no discernable pattern to the results that emerged, no further reports on any single significant moderating paths were made. In summary, adolescent gender was not found to be a moderator of bidirectional links among parent-driven communication strategies, adolescent-driven communication strategies, and adolescent externalizing and internalizing problems. Future studies designed to examine these associations that hypothesize about such gender-specific effects a priori are needed.

## Discussion

The development of adolescent externalizing and internalizing problems may be mitigated by adequate parenting strategies. Existing literature suggests that parents’ asking questions about adolescent whereabouts (i.e., parent solicitation) or communicating rules of behavior (i.e., parent behavioral control) could protect adolescents from maladaptive developmental outcomes (Dishion and McMahon 1998), including externalizing (Pinquart 2017a) and internalizing problems (Pinquart 2017b). However, the development of adolescent psychological problems could be better understood by including adolescent information management, such as disclosure (Stattin and Kerr 2000) and secrecy (Frijns et al. 2010), into models of parenting and adolescent development. Yet, studies including reciprocal associations between parents’ and adolescents’ communication efforts and adolescent psychological problems are scarce, and virtually no investigations capture cultural variation in these processes. Even scarcer are studies in which adolescent disclosure and secrecy are studied as distinct processes of adolescent information management (Villalobos Solís et al. 2015). Therefore, the current study examined how adolescent-reported parent solicitation, parent behavior control, and adolescent disclosure, secrecy, internalizing, and externalizing problems were interrelated over the course of two years in a sample of adolescents from 12 cultures. In addition, to demonstrate whether the potential links between parenting and adolescent psychological functioning are universal or culture specific, the moderating effect of culture on these links was examined.

The results of the current study showed both universal and culture-specific links between parenting and adolescent psychological problems. The universal links that emerged showed positive and reciprocal associations between adolescent secrecy and externalizing problems, positive links from age 13 adolescent disclosure to age 15 parent behavior control and age 15 parent solicitation, and a positive link from age 13 adolescent internalizing problems to age 15 parent behavior control. Some culture-specific links between parent- and adolescent-driven communication efforts and adolescent psychological problems emerged in Chinese, Jordanian, Colombian, Swedish, Kenyan, U.S. Black, and U.S. Latino samples of adolescents. Thus, the current study provides evidence for processes in parent–adolescent communication efforts and adolescent psychological problems both globally and at a specific culture level.

### Links between Parent–Adolescent Communication Efforts and Adolescent Psychological Problems

Based on parenting theories (Soenens et al. 2019), it was expected that parent communication efforts would be negatively related to adolescent psychological problems over time. However, results of this study showed no significant links from parent communication efforts to adolescent psychological problems. In contrast, lower levels of parent behavioral control seem to be predicted by greater adolescent internalizing problems. These null findings are somewhat surprising given that parent behavioral control is usually referred to as a parenting strategy protective of the development of children’s externalizing (Pinquart 2017a) and internalizing problems (Pinquart 2017b). One explanation for the null results may be the developmental stage of the adolescents. Early adolescence is characterized by increased individuation from parents as well as enhanced need of autonomy (Soenens et al. 2019). Such changes are often reflected in decreased levels of parental behavioral control as well as decreases in adolescents’ willingness to conform to rules (Masche 2010) and an increased likelihood of adolescents finding parents’ actions intrusive instead of caring (Hawk et al. 2008). Supporting this hypothesis, the non-significant association between parent-driven communication efforts and adolescent psychological outcomes corroborates other empirical research, which found null effects of parent-driven communication on early adolescents’ delinquency (Keijsers et al. 2010a) and substance use (Kapetanovic et al. 2019b). Another explanation for these null results may be the inclusion of adolescents’ own communication efforts in the model. Preliminary bivariate correlations across the entire sample showed that parent solicitation and behavioral control were both significantly and negatively associated with contemporaneous child externalizing behavior, and parent behavioral control was significantly negatively correlated with subsequent child externalizing behavior (as expected given prior meta-analyses e.g., Pinquart 2017a, 2017b). However, parent communication efforts were non-significant predictors of adolescent psychological problems once adolescents’ own communication efforts were included in the model. The null results align with those seen from other studies that simultaneously assessed adolescents’ own communication efforts and parent-driven communication. In these studies, parent communication efforts do not emerge as significant unique predictors of adolescent problems (see Kapetanovic et al. 2017). Notably, the present results showed that only adolescents’ own communication efforts were related to their psychosocial problems over time. Thus, adolescent-driven communication efforts, or more specifically adolescent secrecy, as opposed to parent-driven communication efforts, seem to be more important predictors of subsequent psychological problems. The current findings extend the classic Stattin and Kerr (2000) work by emphasizing that both parents’ and adolescents’ communication patterns need to be examined simultaneously to grasp associations between parent–adolescent communication and adolescent psychological problems in many cultural contexts.

In addition, most earlier studies (e.g., Kapetanovic et al. 2019a) include adolescent communication effort as a mixture of disclosure and non-secrecy. However, as suggested by scholars (Frijns et al. 2010), this study included adolescent secrecy and disclosure as distinct constructs. Corroborating results from Frijns et al. (2010), adolescent secrecy, but not disclosure, was linked to psychological problems over time. More specifically, adolescent secrecy was bidirectionally associated with higher levels of externalizing problems, meaning that higher levels of secrecy predicted externalizing problems, and higher externalizing problems predicted higher levels of secrecy. Withholding information from parents may be a normative part of the adolescent individuation process linked to more autonomy from parents (Finkenauer et al. 2002). Yet, a consequence of this secrecy may be that parents become less knowledgeable of their adolescents’ whereabouts which may result in fewer opportunities to support and guide their adolescents and more opportunities for adolescents to engage in aggressive or delinquent behavior. To clarify, externalizing problems are marked by lack of self-control and exhibition of rule breaking, aggressive, and delinquent behaviors (Merikangas et al. 2010). Adolescents exhibiting such problems are often met with criticism from peers or adults (Dishion and Patterson 1998). Because adolescents with such problems often lack skills to control their behavior, criticism from others may induce feelings of guilt and shame. As a result, adolescents may withdraw and withhold important information from others, including their parents, so that they may avoid shame and sanctions, and continue to engage in delinquent and externalizing behavior (Dishion and Patterson 1998). Attachment to parents is often considered as a prerequisite for acquiring self-control skills and for positive psychosocial development (Hirschi 1969). Keeping secrets from parents may have an adverse effect on the parent–adolescent relationship, putting a strain on the emotional bond between parents and their adolescent children. In other words, withholding information does not necessarily result in personal autonomy (in a positive sense) as proposed by Finkenauer et al. (2002) for all adolescents, but in some adolescents could lead to alienation from parents, which increases opportunities for unmonitored opportunities for delinquency, affiliation with deviant peers, and consequent externalizing behavior (Dishion and Patterson 1998). In that sense, adolescent secrecy could facilitate a vicious cycle where parents have difficulty reaching out and helping their adolescents to improve their self-control skills, and adolescents demonstrate more externalizing problems.

Moreover, corroborating the findings in other studies (Kapetanovic et al. 2019a), the present results showed that in a cross-cultural sample of adolescents, adolescent disclosure was associated with higher levels of parent behavioral control and solicitation over time. This finding indicates that adolescents’ willingness to communicate with parents may be the driving force in parent–adolescent communication. Sharing information with parents could be interpreted by parents as a sign that their adolescent is open to communicating with their parents, which facilitates opportunities for parents to ask questions and stipulate their own expectations and rules. In other words, adolescents seem to provide opportunities for parents to be engaged in their adolescents’ lives. The word of caution is that the significance levels in links from adolescent disclosure to behavioral control were only marginal (*p* = 0.053–0.059). However, because the findings were in line with other earlier studies, were nearly significant even after controlling for other powerful covariates, and were nearly significant across all 12 cultures studied, they are reported here.

### Are the Links between Parent–Adolescent Communication Efforts and Adolescent Psychological Problems Moderated by Culture?

An important question in parenting research is whether parenting effects on children’s and adolescents’ psychosocial development are universal or differ by culture. The current study addressed this question by finding that, in addition to the universal effects reported above, there are some culture-specific links among parent–adolescent communication and adolescent psychological problems. First, adolescent internalizing problems were prospectively associated with less disclosure, while adolescent secrecy was prospectively associated with greater internalizing problems in Jordan and Colombia. Furthermore, a negative link from internalizing problems to disclosure also emerged in the Chinese sample, and a negative link from externalizing problems to disclosure emerged in the Swedish and Kenyan samples of adolescents. These findings indicate that the relation between adolescent communication efforts and internalizing problems in Jordanian and Colombian adolescents was negative and intertwined such that adolescents who experienced internalizing problems disclosed less over time, while their secrecy toward parents resulted in more internalizing problems over time. In both Jordanian and Colombian culture, respect and guidance (Oweis et al. 2012) as well as loyalty and attachment between family members (Di Giunta et al. 2011) are especially strongly valued. Consequently, non-normative lack of communication between parents and adolescents in these cultures might be especially deleterious to both parent–adolescent communication and adolescent internalizing symptoms, given the central role family relationships play in both Jordan and Colombia. When adolescents share information with their parents, parents are given opportunities to provide social support, which is one of the most powerful protective factors that buffers adolescents against internalizing problems (Hamza and Willoughby 2011). By contrast, when adolescents experience internalizing problems they tend to withdraw from others, including parents (Rothenberg et al. 2018) and therefore disclose less (Hamza and Willoughby 2011), which makes it harder for parents to be involved and provide such preventative social support. In cultures such as Jordan and Kenya, this lack of social support might be especially detrimental because family relationships are so highly valued in both cultures.

It is however somewhat surprising that similar links were not found among Western adolescents given the widely demonstrated link between adolescent-driven communication efforts and psychological problems (e.g., Racz and McMahon 2011). Then again, earlier studies with Western adolescents predominantly measured adolescent disclosure as a mixture of disclosure and secrecy (Stattin and Kerr 2000) (rather than separating these two constructs as proposed by Frijns et al. 2010) and often investigated externalizing (e.g., Racz and McMahon 2011) rather than internalizing problems. One exception is a study by Hamza and Willoughby (2011) where a negative link from adolescent depressive symptoms to disclosure was found among Canadian mid-adolescents and a study by Jäggi et al. (2016) where a negative link between depressive symptoms to secrecy was found among US Black preadolescents. Thus, specific prospective links between adolescent disclosure, secrecy, and internalizing among early adolescents have rarely been studied prior to the present investigation, and less is known about these links worldwide. Perhaps such links are less common than intuitively expected. The largely non-significant link between parent–adolescent communication and internalizing problems could also be a matter of how adolescent disclosure, in particular, is conceptualized. Scholars suggest that differentiating between routine disclosure (i.e., sharing of information about one’s whereabouts, as measured here) and self-disclosure (i.e., sharing of deeper and more intimate information) is imperative when studying adolescent information management and the potential links to adolescent developmental trajectories (Tilton-Weaver et al. 2014). Although adolescent disclosure indeed is related to emotional connectedness between parents and their adolescents (Kapetanovic et al. 2019c), it is possible that measures of more specific and personal disclosure would provide more insight in the investigation of potential correlates of internalizing problems. More studies where adolescent internalizing problems and disclosure (routine or personal) and secrecy are studied are needed to gain better understanding of the processes between adolescent communication efforts and internalizing problems.

Furthermore, a negative link from internalizing problems to disclosure emerged in the Chinese sample, and a negative link from externalizing problems to disclosure emerged in the Swedish and Kenyan samples of adolescents. These findings indicate that the Chinese adolescents who were experiencing internalizing problems, and Swedish and Kenyan adolescents who were experiencing externalizing problems, disclosed less information to their parents over time. Another recent study (Lansford et al. 2018a) found that China had the second-lowest overall levels of adolescent internalizing behaviors, and both Sweden and Kenya had some of the lowest overall levels of externalizing behaviors. It is possible that when adolescents in these cultures experience psychological problems that are especially rare, they may be even more disturbed or ashamed of such problems, and therefore even less likely to discuss such problems with their parents, compared to adolescents in other cultures where such problems are more normative. Therefore, cultural norms about the experiences of such problems (i.e., that they rarely occur) may make the link between experiencing behavior problems and avoiding disclosure even stronger. Additionally, in China, Colombia, Thailand and U.S. Black samples, externalizing problems predicted lower levels of parent behavior control over time. In all of these cultures, harmony with one’s family and society is especially highly prized (Lansford et al. 2018a). Therefore, it may be that in each of these cultures, as adolescents experience more externalizing problems, parents provide adolescents with less control and more autonomy to preserve family harmony and avoid conflict over the externalizing behavior.

Finally, some parent effects on parent–adolescent communication emerged in the Colombian, U.S. Black, and U.S. Latino samples. Specifically, a positive link from parent solicitation to adolescent disclosure was discovered in the samples of Black and Latino adolescents, which corroborates findings in other studies using U.S. Black (Bumpus and Rodgers 2009) and U.S. Latino samples (Fernandez et al. 2018) where within-time correlations between parental solicitation and adolescent disclosure were found. In both U.S. Black (McLoyd et al. 2019) and Latino (Halgunseth 2019) cultures, the values of family cohesion and respect are heavily emphasized. For example, in Latino cultures, the concept of “*familismo*” represents the strong loyalty and attachment between different family members, including respectful and considerate behavior toward authorities, such as parents. It is therefore possible that parent solicitation and adolescent disclosure are especially likely to be linked in U.S. Black and Latino cultures due to the high emphasis on family relationships. Another parent effect was discovered in Colombian and U.S. Black samples where a negative link between parent behavioral control and adolescent disclosure emerged. Parent behavioral control was expected to be linked to higher disclosure because the structure that parents offer through their behavioral expectations is relevant for adolescents’ need for competence and, in turn, parent–adolescent communication (Soenens et al. 2019). Whether parents impose high, moderate, or low levels of behavioral expectations is highly important for adolescent adjustment (Harris-McKoy 2016). Prior work examining parent behavioral control in data used in the current study confirmed that parents in both the U.S. Black and Colombian samples demonstrate significantly higher behavioral control than the sample as a whole (Rothenberg et al. 2019). Given that parent behavioral control has been linked to increased feelings of being overly controlled (Kapetanovic et al. 2017), the high levels of parent behavioral control experienced by adolescents in these cultures may make it especially likely that adolescents feel overcontrolled, and therefore unlikely to disclose. Future studies should also include adolescent interpretations of parents’ behavioral control in studies linking parent–adolescent communication and adolescent psychological problems.

Given the lack of studies including adolescents from non-western societies, the current study takes an important step towards understanding processes in parent–adolescent communication and adolescent psychological problems across cultures. However, one limitation of the study is that the adolescents are representative of the locales, but not wider countries, from which they were drawn, so national differences cannot be inferred from the current work. Future work could therefore build on this investigation in nationally representative samples. An additional limitation is that the data only consisted of adolescents’ self-reports. Thus, associations in this study could be inflated due to response bias or common method variance. Studies indicate that parents and adolescents have different views of their interactions (de Los Reyes 2011) which is why future studies should also include parents’ perceptions of parent–adolescent communication. Additionally, though the current sample is relatively large (*n* = 1087), culture-specific sample sizes are rather small (with *ns* around 100 in each culture). Therefore, it is possible that the study was underpowered to detect all culture-specific study effects. Consequently, replication and expansion of these findings in larger culture-specific samples is warranted. Also, although cross-lagged models are informative of multiple developmental processes (Meeus 2016), two-wave models do not explicate whether the links between the constructs are stable or changing over time. A three-wave longitudinal design would more thoroughly capture changing processes in the transactional linkages and disentangle whether the relations between constructs stabilize or change over time (Ployhart and Vandenberg 2010). Thus, future studies should include several timepoints to understand the change in long-term developmental processes between parent–adolescent communication and adolescent psychological health. In addition, because parent–adolescent interactions are not stable, but may fluctuate from day to day, another recommendation is to study links between parent–adolescent communication and adolescent psychological problems on a shorter time scale, such as on a weekly or daily basis (Villalobos Solís et al. 2015). Combining these methodologies would lend insight into short-term as well as long-term processes in parent–adolescent communication and development of adolescent psychological problems.

## Conclusion

Parent–adolescent communication is one of the key mechanisms linking parent–adolescent relationships and the development of psychological problems in adolescence (Stattin and Kerr 2000). Such communications can be parent-driven (parent behavioral control and solicitation) or adolescent-driven (disclosure and secrecy). Most existing studies do not separate adolescent disclosure from secrecy (as suggested by scholars; Finkenauer et al. 2002), often conduct research on western samples of adolescents, and do not examine processes longitudinally. The current study filled these gaps by examining how parent solicitation, behavioral control, adolescent disclosure, secrecy, internalizing, and externalizing problems are interrelated over the course of two years and testing whether the potential links between parenting and adolescent psychological functioning are moderated by culture. The results revealed that adolescents’ own management of information (particularly via secrecy) is intertwined with their psychological problems both universally and on a culture-specific level. Notably, adolescent withholding of information from their parents predicted more externalizing problems over time, and adolescent externalizing problems predicted more secrecy from parents over time. In some cultures (i.e., Colombia and Jordan), withholding information from parents was also linked to internalizing problems over time. These findings underscore the importance of adolescents’ own information management, and specifically their secrecy toward parents, in predicting adolescent psychological functioning. Put simply, across cultures, adolescent secrecy towards parents could be a signal for future adolescent psychological difficulties. The findings further reveal that adolescent information management also appears to influence subsequent parent-driven communication efforts across cultural settings. Specifically, in all cultures, greater adolescent disclosure predicted greater parent control and solicitation two years later. In the US Black (for control and solicitation), US Latino (for solicitation), and Colombian (for control) samples, these links were reciprocal. Taken together, the findings reveal that although parents play an important role in fostering parent–adolescent communication, the parent–adolescent communication process seems to be adolescent-driven, rather than parent-driven in cultures around the world. To understand adolescent psychological development, adolescent agency, in this case expressed by management of information via disclosure and secrecy, should be emphasized in parenting studies as well as implications for practices. In particular, addressing adolescent secrecy toward parents could be key in preventing externalizing and internalizing problems in many cultural contexts worldwide.

## Supplementary information

Supplementary Information

## References

[CR1] Achenbach TM, Rescorla LA (2001). Manual for the ASEBA school-age forms & profiles.

[CR2] Ahmad Ikhlas, Smetana Judith G., Klimstra Theo (2014). Maternal Monitoring, Adolescent Disclosure, and Adolescent Adjustment Among Palestinian Refugee Youth in Jordan. Journal of Research on Adolescence.

[CR3] Akcinar Berna, Baydar Nazli (2014). Parental control is not unconditionally detrimental for externalizing behaviors in early childhood. International Journal of Behavioral Development.

[CR5] Bornstein Marc H. (2012). Cultural Approaches to Parenting. Parenting.

[CR6] Bornstein MH, Jager J, Steinberg LD, Weiner I, Lerner RM, Easterbrooks MA, Mistry J (2013). Adolescents, parents, friends/peers: a relationships model. Handbook of psychology, Vol. 6: Developmlental psychology.

[CR7] Bornstein Marc H., Putnick Diane L., Lansford Jennifer E. (2011). Parenting Attributions and Attitudes in Cross-Cultural Perspective. Parenting.

[CR8] Bumpus Matthew F., Rodgers Kathleen Boyce (2009). Parental Knowledge and Its Sources. Journal of Family Issues.

[CR9] Darling Nancy, Steinberg Laurence (1993). Parenting style as context: An integrative model. Psychological Bulletin.

[CR10] De Los Reyes Andres (2011). Introduction to the Special Section: More Than Measurement Error: Discovering Meaning Behind Informant Discrepancies in Clinical Assessments of Children and Adolescents. Journal of Clinical Child & Adolescent Psychology.

[CR11] Di Giunta L, Uribe Tirado LM, Araque Márquez LA (2011). Attributions and attitudes of mothers and fathers in Colombia. Parenting: Science and Practice.

[CR12] Dishion, T. J., & McMahon, R. J. (1998). Parental monitoring and the prevention of child and adolescent problem behavior: a conceptual and empirical formulation. *Clinical Child and Family Psychology Review*. 10.1023/A:1021800432380.pdf. 10.1023/a:1021800432380 11324078

[CR13] Enders CK (2010). Applied missing data analysis.

[CR14] Erkut Sumru (2010). Developing Multiple Language Versions of Instruments for Intercultural Research. Child Development Perspectives.

[CR15] Fernandez Alejandra, Loukas Alexandra, Pasch Keryn E. (2018). Examining the Bidirectional Associations between Adolescents’ Disclosure, Parents’ Solicitation, and Adjustment Problems among Non-Hispanic White and Hispanic Early Adolescents. Journal of Youth and Adolescence.

[CR16] Finkenauer Catrin, Engels Rutger C. M. E., Meeus Wim (2002). Keeping Secrets from Parents: Advantages and Disadvantages of Secrecy in Adolescence. Journal of Youth and Adolescence.

[CR17] Fletcher Anne C., Steinberg Laurence, Williams-Wheeler Meeshay (2004). Parental Influences on Adolescent Problem Behavior: Revisiting Stattin and Kerr. Child Development.

[CR18] Frijns T, Keijsers L, Branje S, Meeus W (2010). What parents don’t know and how it may affect their children: qualifying the disclosure-adjustment link. Journal of Adolescence.

[CR19] Garthe Rachel C., Sullivan Terri, Kliewer Wendy (2014). Longitudinal Relations Between Adolescent and Parental Behaviors, Parental Knowledge, and Internalizing Behaviors Among Urban Adolescents. Journal of Youth and Adolescence.

[CR20] Halgunseth Linda C. (2019). Latino and Latin American Parenting. Handbook of Parenting.

[CR21] Hamza Chloe A., Willoughby Teena (2010). Perceived Parental Monitoring, Adolescent Disclosure, and Adolescent Depressive Symptoms: A Longitudinal Examination. Journal of Youth and Adolescence.

[CR22] Harris-McKoy DeAnna (2016). Adolescent Delinquency: Is Too Much or Too Little Parental Control a Problem?. Journal of Child and Family Studies.

[CR23] Hawk Skyler T., Hale William W., Raaijmakers Quinten A. W., Meeus Wim (2008). Adolescents' Perceptions of Privacy Invasion in Reaction to Parental Solicitation and Control. The Journal of Early Adolescence.

[CR24] Hessel Heather, He Yaliu, Dworkin Jodi (2016). Paternal Monitoring: The Relationship Between Online and In-Person Solicitation and Youth Outcomes. Journal of Youth and Adolescence.

[CR25] Hirschi, T. (1969). A control theory of delinquency. In P. W. Frank, D. M. Marilyn (Eds), *Criminology theory: selected classic readings*. (pp. 289–305). Cincinnati, OH: Anderson Publishing.

[CR26] Jäggi Lena, Drazdowski Tess K., Kliewer Wendy (2016). What parents don't know: Disclosure and secrecy in a sample of urban adolescents. Journal of Adolescence.

[CR27] Kapetanovic Sabina, Boele Savannah, Skoog Therése (2019). Parent-Adolescent Communication and Adolescent Delinquency: Unraveling Within-Family Processes from Between-Family Differences. Journal of Youth and Adolescence.

[CR28] Kapetanovic Sabina, Bohlin Margareta, Skoog Therese, Gerdner Arne (2017). Structural relations between sources of parental knowledge, feelings of being overly controlled and risk behaviors in early adolescence. Journal of Family Studies.

[CR29] Kapetanovic Sabina, Skoog Therése, Bohlin Margareta, Gerdner Arne (2019). Does one Size Fit All?—Linking Parenting With Adolescent Substance Use and Adolescent Temperament. Journal of Research on Adolescence.

[CR30] Kapetanovic Sabina, Skoog Therése, Bohlin Margareta, Gerdner Arne (2019). Aspects of the parent–adolescent relationship and associations with adolescent risk behaviors over time. Journal of Family Psychology.

[CR31] Keijsers Loes, Branje Susan J. T., VanderValk Inge E., Meeus Wim (2010). Reciprocal Effects Between Parental Solicitation, Parental Control, Adolescent Disclosure, and Adolescent Delinquency. Journal of Research on Adolescence.

[CR32] Keijsers Loes, Branje Susan J. T., Frijns Tom, Finkenauer Catrin, Meeus Wim (2010). Gender differences in keeping secrets from parents in adolescence. Developmental Psychology.

[CR33] Kerr Margaret, Stattin Håkan (2000). What parents know, how they know it, and several forms of adolescent adjustment: Further support for a reinterpretation of monitoring. Developmental Psychology.

[CR34] Kerr Margaret, Stattin Håkan, Burk William J. (2010). A Reinterpretation of Parental Monitoring in Longitudinal Perspective. Journal of Research on Adolescence.

[CR35] Kline RB (2011). Principles and practices of structural equation modeling.

[CR36] Kuczynski, L., & Mol De, J. (2015). Dialectical models of socialization. In W. F. Overton & P. C. M. Molenaar (Eds), *Theory and Method. Volume 1 of the**Handbook of child psychology and developmental science.* 7th ed., Vol 1 (pp. 326–368). Hoboken, NJ: Wiley.

[CR37] Deater-Deckard Kirby, Godwin Jennifer, Lansford Jennifer E., Bacchini Dario, Bombi Anna Silvia, Bornstein Marc H., Chang Lei, Di Giunta Laura, Dodge Kenneth A., Malone Patrick S., Oburu Paul, Pastorelli Concetta, Skinner Ann T., Sorbring Emma, Steinberg Laurence, Tapanya Sombat, Alampay Liane Peña, Uribe Tirado Liliana Maria, Zelli Arnaldo, Al-Hassan Suha M. (2018). Within- and between-person and group variance in behavior and beliefs in cross-cultural longitudinal data. Journal of Adolescence.

[CR38] Lansford Jennifer E., Rothenberg W. Andrew, Jensen Todd M., Lippold Melissa A., Bacchini Dario, Bornstein Marc H., Chang Lei, Deater-Deckard Kirby, Di Giunta Laura, Dodge Kenneth A., Malone Patrick S., Oburu Paul, Pastorelli Concetta, Skinner Ann T., Sorbring Emma, Steinberg Laurence, Tapanya Sombat, Uribe Tirado Liliana Maria, Alampay Liane Peña, Al-Hassan Suha M. (2018). Bidirectional Relations Between Parenting and Behavior Problems From Age 8 to 13 in Nine Countries. Journal of Research on Adolescence.

[CR39] Lionetti, F., Keijsers, L., Dellagiulia, A., & Pastore, M. (2016). Evidence of factorial validity of parental knowledge, control and solicitation, and adolescent disclosure scales: when the ordered nature of Likert scales matters. *Frontiers in Psychology.*10.3389/fpsyg.2016.00941. 10.3389/fpsyg.2016.00941 PMC4916221 27445909

[CR40] Leaper C, Farkas T, Grusec JE, Hastings PD (2015). The socialization of gender during childhood and adolescence. Handbook of socialization: theory and research.

[CR42] McLoyd, V. C., Hardaway, C. R., & Jocson, R. M. (2019). African American parenting. In M. H. Bornstein (Ed.), *Handbook of parenting: children and parenting.* 3rd ed., Vol 1 (pp. 57–107). New York: Routledge.

[CR43] Marshall Sheila K., Tilton-Weaver Lauree C., Bosdet Lara (2005). Information management: Considering adolescents’ regulation of parental knowledge. Journal of Adolescence.

[CR44] Masche J. Gowert (2010). Explanation of normative declines in parents' knowledge about their adolescent children. Journal of Adolescence.

[CR45] Meeus Wim (2016). Adolescent psychosocial development: A review of longitudinal models and research. Developmental Psychology.

[CR46] Merikangas Kathleen Ries, He Jian-ping, Burstein Marcy, Swanson Sonja A., Avenevoli Shelli, Cui Lihong, Benjet Corina, Georgiades Katholiki, Swendsen Joel (2010). Lifetime Prevalence of Mental Disorders in U.S. Adolescents: Results from the National Comorbidity Survey Replication–Adolescent Supplement (NCS-A). Journal of the American Academy of Child & Adolescent Psychiatry.

[CR47] Morawska AL, Winter MR, Sanders MR (2009). Parenting knowledge and its role in the prediction of dysfunctional parenting and disruptive child behaviour. Child: Care, Health and Development.

[CR48] Muthén LK, Muthén BO (1998). Mplus user’s guide.

[CR70] Oweis A, Gharaibeh M, Maaitah R, Gharaibeh H, Obeisat S (2012). Parenting from a Jordanian perspective: findings from a qualitative study. Journal of Nursing Scholarship.

[CR49] Pinquart, M. (2017a). Associations of parenting dimensions and styles with externalizing problems of children and adolescents: an updated meta-analysis. *Developmental Psychology*. 10.1037/dev0000295.supp. 10.1037/dev0000295 28459276

[CR50] Pinquart M (2017). Associations of parenting dimensions and styles with internalizing symptoms in children and adolescents: a meta-analysis. Marriage & Family Review.

[CR51] Ployhart Robert E., Vandenberg Robert J. (2009). Longitudinal Research: The Theory, Design, and Analysis of Change. Journal of Management.

[CR52] Putnick Diane L., Bornstein Marc H., Lansford Jennifer E., Chang Lei, Deater-Deckard Kirby, Di Giunta Laura, Gurdal Sevtap, Dodge Kenneth A., Malone Patrick S., Oburu Paul O., Pastorelli Concetta, Skinner Ann T., Sorbring Emma, Tapanya Sombat, Tirado Liliana Maria Uribe, Zelli Arnaldo, Alampay Liane Peña, Al-Hassan Suha M., Bacchini Dario, Bombi Anna Silvia (2012). Agreement in Mother and Father Acceptance-Rejection, Warmth, and Hostility/Rejection/ Neglect of Children Across Nine Countries. Cross-Cultural Research.

[CR53] Racz Sarah Jensen, McMahon Robert J. (2011). The Relationship Between Parental Knowledge and Monitoring and Child and Adolescent Conduct Problems: A 10-Year Update. Clinical Child and Family Psychology Review.

[CR54] Reinke Wendy M., Eddy J. Mark, Dishion Thomas J., Reid John B. (2012). Joint Trajectories of Symptoms of Disruptive Behavior Problems and Depressive Symptoms During Early Adolescence and Adjustment Problems During Emerging Adulthood. Journal of Abnormal Child Psychology.

[CR55] Reitz E., Deković M., Meijer A. M. (2005). The Structure and Stability of Externalizing and Internalizing Problem Behavior During Early Adolescence. Journal of Youth and Adolescence.

[CR56] Rote Wendy M., Smetana Judith G. (2015). Beliefs About Parents' Right to Know: Domain Differences and Associations With Change in Concealment. Journal of Research on Adolescence.

[CR57] Rothenberg W. Andrew, Hussong Andrea M., Chassin Laurie (2018). Intergenerational continuity in high-conflict family environments: Investigating a mediating depressive pathway. Developmental Psychology.

[CR58] Rothenberg WA, Lansford JE, Alampay LP, Al-Hassan SM, Bacchini D, Bornstein MH, Yotanyamaneewong S (2019). Examining effects of mother and father warmth and control on child externalizing and internalizing problems from age 8 to 13 in nine countries. Development and Psychopathology.

[CR59] Sameroff Arnold (2010). A Unified Theory of Development: A Dialectic Integration of Nature and Nurture. Child Development.

[CR60] Smetana Judith G. (2008). "It’s 10 O’Clock: Do You Know Where Your Children Are?" Recent Advances in Understanding Parental Monitoring and Adolescents’ Information Management. Child Development Perspectives.

[CR61] Smetana Judith G (2017). Current research on parenting styles, dimensions, and beliefs. Current Opinion in Psychology.

[CR63] Soenens, B, Vansteenkiste, M., & Beyers, W. (2019). Parenting adolescents. In M. H. Bornstein (Ed.), Handbook of parenting: children and parenting. 3rd ed., Vol 1. (pp. 101–167). New york, NY: Routledge.

[CR64] Stattin Hakan, Kerr Margaret (2000). Parental Monitoring: A Reinterpretation. Child Development.

[CR65] Tilton-Weaver Lauree (2013). Adolescents’ Information Management: Comparing Ideas About Why Adolescents Disclose to or Keep Secrets from Their Parents. Journal of Youth and Adolescence.

[CR66] Tilton-Weaver Lauree C., Marshall Sheila K., Darling Nancy (2013). What's in a Name? Distinguishing Between Routine Disclosure and Self-Disclosure. Journal of Research on Adolescence.

[CR67] Villalobos Solís Myriam, Smetana Judith G., Comer Jessamy (2015). Associations among solicitation, relationship quality, and adolescents' disclosure and secrecy with mothers and best friends. Journal of Adolescence.

[CR69] Yarnell Lisa M., Sargeant Marsha N., Prescott Carol A., Tilley Jacqueline L., Farver Jo Ann M., Mednick Sarnoff A., Venables Peter H., Raine Adrian, Luczak Susan E. (2013). Measurement Invariance of Internalizing and Externalizing Behavioral Syndrome Factors in a Non-Western Sample. Assessment.

