# Supplementary material for: Cross-Cultural Examination of Links between Parent–Adolescent Communication and Adolescent Psychological Problems in 12 Cultural Groups
Source: J Youth Adolesc. 2020 Mar 12;49(6):1225–44. doi: 10.1007/s10964-020-01212-2 (PMC7237396; doi:10.1007/s10964-020-01212-2)
Supplement: Supplementary file 1 — Supplementary Information [file 10964_2020_1212_MOESM1_ESM.docx]

Supplementary Table 1. Overview of the Items in Parent Behavior Control and Solicitation, and Adolescent Disclosure and Secrecy Scales

| Scale | Items |
| --- | --- |
| Parent Behavior Control | Must you have your parents' permission before you go out during weeknights?  If you go out on a Saturday evening, must you inform your parents beforehand about who will be along as well as where you are?  If you have been out past curfew, do your parents require that you explain why and tell who you were with?  Do your parents demand that they know where you are in the evenings, who you are going to be with, and what you are going to do?  Must you ask your parents before you can make plans with friends about what you will do on Saturday night?  Do your parents require that you tell them how you spend your money? |
| Parent Solicitation | How often do your parents talk to your friends when they come over to your house?  During the past month, how often have your parents initiated a conversation with you about your free time?  How often do your parents ask you what happened during your free time?  How often do your parents ask you to sit and tell them what happened at school on a regular school day? |
| Adolescent Disclosure | Do you spontaneously tell your parents about your friends?  How often do you usually want to tell your parents about school?  Do you like to tell your parents where you went and what you did during the evening? |
| Adolescent Secrecy | Do you keep a lot of secrets from your parents about what you do during your free time?  Do you hide a lot from your parents about what you do during nights and weekends? |
